# Um Passo à Frente na Avaliação da Força de Preensão Manual na Insuficiência Cardíaca

**DOI:** 10.36660/abc.20250579

**Published:** 2025-10-28

**Authors:** Evandro José Cesarino, Marildes Luiza de Castro, Regina Célia Garcia de Andrade, Carolina Baraldi Araujo Restini

**Affiliations:** 1 Universidade de São Paulo-Faculdade de Ciências Farmacêuticas de Ribeirão Preto Ribeirão Preto SP Brasil Universidade de São Paulo-Faculdade de Ciências Farmacêuticas de Ribeirão Preto, Ribeirão Preto, SP – Brasil; 2 Associação Ribeirãopretana de Ensino, Pesquisa e Assistência ao Hipertenso Ribeirão Preto SP Brasil Associação Ribeirãopretana de Ensino, Pesquisa e Assistência ao Hipertenso (AREPAH), Ribeirão Preto, SP – Brasil; 3 Faculdade de Medicina da Universidade Federal de Minas Gerais Hospital das Clínicas Belo Horizonte MG Brasil Hospital das Clínicas, Faculdade de Medicina da Universidade Federal de Minas Gerais (UFMG), Belo Horizonte, MG – Brasil; 4 Michigan State University Pharmacology and Toxicology Dept. College of Osteopathic Medicine Michigan EUA College of Osteopathic Medicine, Pharmacology and Toxicology Dept., Michigan State University, Michigan – EUA

**Keywords:** Força da Mão, Valores de Referência, Insuficiência Cardíaca, Insuficiência Cardíaca Sistólica, Insuficiência Cardíaca Diastólica

A insuficiência cardíaca (IC) afeta milhões de pessoas em todo o mundo, causando morbidade e mortalidade significativas. A capacidade funcional, um determinante crítico do prognóstico e da qualidade de vida, é frequentemente prejudicada em pacientes com IC devido à fraqueza e fragilidade muscular, afetando até 40% dessa população.^1,2^ O manuscrito "Força de Preensão Manual na Insuficiência Cardíaca: Estabelecendo uma Equação de Referência" apresenta um esforço pioneiro ao desenvolver uma equação de referência validada para prever a força de preensão manual (FPM) em pacientes com IC, abordando lacunas em ferramentas de avaliação clínica adaptadas a esse grupo.^3^ Este minieditorial avalia as contribuições, os pontos fortes, as limitações e as implicações deste estudo para a prática clínica.

## Importância da força de preensão manual na IC

A FPM é uma medida simples e não invasiva da força muscular, correlacionando-se com a condição física e considerada um marcador prognóstico na IC.^4,5^ Uma revisão sistemática de 7.350 pacientes com IC revelou que uma redução de 1 kg na FPM aumenta o risco de mortalidade em 8% (RR 1,08, IC 95% 1,05–1,11).^5^ Ao contrário de medidas complexas, como o teste de exercício cardiopulmonar, a FPM é de baixo custo, acessível e viável em todos os cenários clínicos, tornando-se uma ferramenta direta para avaliar a fragilidade e orientar a reabilitação.^6,7^ No entanto, as equações de referência de FPM existentes são derivadas principalmente de populações saudáveis, falhando em levar em conta fatores específicos da IC, como débito cardíaco (DC) reduzido e atrofia muscular, levando a conclusões imprecisas.^8^ O desenvolvimento do manuscrito de uma equação de FPM específica para IC representa um avanço oportuno.

## Pontos fortes do estudo

O estudo transversal envolvendo 274 pacientes (18–79 anos) com IC estável (174 no braço de derivação e 100 no braço de validação) foi adequado para estabelecer uma equação de referência.^3^ Os autores empregaram metodologia rigorosa, usando um dinamômetro com protocolos padronizados para garantir medições confiáveis da FPM (por exemplo, três contrações máximas com a mão dominante).^9^ O modelo de regressão multivariada, incorporando idade, sexo, altura, circunferência da panturrilha e classe funcional da New York Heart Association (NYHA), explica 57% da variância da FPM (R²=0,578), com boa concordância preditiva (CCI=0,79, IC 95% 0,69–0,86) na coorte de validação.^3^ Essas variáveis se alinham com determinantes conhecidos da força muscular: idade e sexo refletem declínio neuromuscular e diferenças de gênero,^10^ altura se correlaciona com massa óssea e muscular,^11^ circunferência da panturrilha pode indicar massa corporal magra e classe funcional da NYHA reflete a gravidade da IC. A ligeira subestimação da equação (resíduo médio: 0,68±8,93 kg) é comparável aos resíduos em estudos de populações saudáveis, sugerindo precisão aceitável.

## Limitações e Considerações

Apesar de seus pontos fortes, o estudo tem limitações. A amostra de conveniência de um único hospital público brasileiro pode limitar a generalização devido a diferenças étnicas, socioeconômicas e de acesso à saúde que podem afetar a FPM. A ausência de dados longitudinais restringe os insights sobre as mudanças na FPM ao longo do tempo ou sua utilidade prognóstica na IC descompensada.^3^ A dependência da equação na circunferência da panturrilha, embora relevante, pode ser menos prática em cenários sem antropometria treinada, e a subjetividade da classe funcional da NYHA pode introduzir variabilidade.^12^ A validação externa em diversas populações com IC (diferentes etnias e etiologias de IC) é necessária para confirmar sua aplicabilidade prática definitivamente.^3^ Comparado a equações de população saudável (por exemplo, Novaes et al., R² = 0,677 para a mão dominante),^13^ o R² menor da equação de IC sugere que fatores não medidos (estado nutricional, capacidade de exercício, etc.) podem influenciar a FPM na IC.

## Implicações clínicas e educacionais

Esta equação fornece aos médicos uma ferramenta prática para avaliar a FPM em pacientes com IC, permitindo a identificação de fragilidade e fraqueza muscular para intervenções personalizadas, como treinamento físico.^14^ A equação ressalta a importância de ferramentas específicas para cada condição, melhorando a compreensão do impacto da IC na condição física e complementando as discussões sobre o manejo da IC, destacando o papel da avaliação funcional no prognóstico.^15^

## Direções futuras

Estudos futuros devem validar a equação em populações diversas e explorar seu valor prognóstico em cenários longitudinais. A incorporação de variáveis adicionais (estado nutricional, fração de ejeção, etc.) pode melhorar o valor preditivo. A integração da FPM às avaliações de rotina da IC, juntamente com ferramentas como o teste de caminhada de 6 minutos, pode aprimorar a estratificação do risco cardiovascular e personalizar o tratamento.^5^

## Conclusão

A equação de referência FPM proposta para pacientes com IC representa um passo significativo em direção à avaliação funcional personalizada. Apesar de suas limitações, a metodologia e a relevância clínica do estudo a tornam uma ferramenta simples e valiosa para clínicos e educadores. À medida que o manejo da IC evolui, essa equação pode orientar o rastreamento e a reabilitação da fragilidade, melhorando, em última análise, os resultados funcionais dos pacientes.
