# Supplementary material for: Um Passo à Frente na Avaliação da Força de Preensão Manual na Insuficiência Cardíaca
Source: Arq Bras Cardiol. 2025 Oct 28;122(9):e20250579. [Article in Portuguese] doi: 10.36660/abc.20250579 (PMC12671602; doi:10.36660/abc.20250579)
Supplement: Translation

# Translation: A Step Forward in the Assessment of Handgrip Strength in Heart Failure

**DOI:** 10.36660/abc.20250579i

**Authors:** Evandro José Cesarino, Marildes Luiza de Castro, Regina Célia Garcia de Andrade, Carolina Baraldi Araujo Restini

**Affiliations:** 1 Ribeirão Preto SP Brasil Universidade de São Paulo-Faculdade de Ciências Farmacêuticas de Ribeirão Preto, Ribeirão Preto, SP – Brasil; 2 Ribeirão Preto SP Brasil Associação Ribeirãopretana de Ensino, Pesquisa e Assistência ao Hipertenso (AREPAH), Ribeirão Preto, SP – Brasil; 3 Belo Horizonte MG Brasil Hospital das Clínicas, Faculdade de Medicina da Universidade Federal de Minas Gerais (UFMG), Belo Horizonte, MG – Brasil; 4 Michigan EUA College of Osteopathic Medicine, Pharmacology and Toxicology Dept., Michigan State University, Michigan – EUA

**Keywords:** Hand Strength, Reference Values, Heart Failure, Systolic Heart Failure, Diastolic Heart Failure

Heart failure (HF) affects millions of people worldwide, causing significant morbidity and mortality. Functional capacity, a critical determinant of prognosis and quality of life, is often impaired in HF patients due to muscle weakness and frailty, affecting up to 40% of this population.^1,2^ The manuscript Handgrip Strength in Heart Failure: Establishing a Reference Equation presents a pioneering effort by developing a validated reference equation to predict handgrip strength (HGS) in HF patients, addressing gaps in clinical assessment tools tailored to this group.^3^ This mini-editorial evaluates the contributions, strengths, limitations, and implications of this study for clinical practice.

## Significance of Handgrip Strength in HF

HGS is a simple, non-invasive measure of muscle strength, correlating with physical condition and considered a prognostic marker in HF.^4,5^ A systematic review of 7,350 HF patients revealed that a 1 kg reduction in HGS increases mortality risk by 8% (RR 1.08, 95% CI 1.05–1.11).^5^ Unlike complex measures, such as cardiopulmonary exercise testing, HGS is low-cost, accessible, and feasible in all clinical settings, making it a straightforward tool to assess frailty and guide rehabilitation.^6,7^ However, existing HGS reference equations are mainly derived from healthy populations, failing to account for HF-specific factors, such as reduced cardiac output (CO) and muscle atrophy, leading to inaccurate conclusions.^8^ The manuscript's development of an HF-specific HGS equation represents a timely advance.

## Strengths of the study

The cross-sectional study involving 274 patients (18–79 years) with stable HF (174 in the derivation arm and 100 in the validation arm) was adequate for establishing a reference equation.^3^ The authors employed rigorous methodology, using a dynamometer with standardized protocols to ensure reliable HGS measurements (e.g., three maximum contractions with the dominant hand).^9^ The multivariate regression model, incorporating age, sex, height, calf circumference, and New York Heart Association (NYHA) functional class, explains 57% of HGS variance (R²=0.578), with good predictive agreement (ICC=0.79, 95% CI 0.69–0.86) in the validation cohort.^3^ These variables align with known determinants of muscle strength: age and sex reflect neuromuscular decline and gender diferences,^10^ height correlates with bone and muscle mass,^11^ calf circumference may indicate lean body mass, and NYHA functional class reflects HF severity. The equation's slight underestimation (mean residual: 0.68±8.93 kg) is comparable to residuals in studies of healthy populations, suggesting acceptable accuracy.

## Limitations and considerations

Despite its strengths, the study has limitations. The convenience sample from a single Brazilian public hospital may limit generalizability due to ethnic, socioeconomic, and healthcare access differences that can affect HGS. The absence of longitudinal data restricts insights into HGS changes over time or its prognostic utility in decompensated HF.^3^ The equation's reliance on calf circumference, while relevant, may be less practical in settings without trained anthropometrics, and the subjectivity of the NYHA functional class may introduce variability.^12^ External validation in diverse HF populations (different ethnicities and HF etiologies) is needed to confirm its practical applicability definitively.^3^ Compared to healthy population equations (e.g., Novaes et al., R²=0.677 for the dominant hand),^13^ the lower R² of the HF equation suggests unmeasured factors (nutritional status, exercise capacity, etc.) may influence HGS in HF.

## Clinical and educational implications

This equation provides clinicians with a practical tool to assess HGS in HF patients, enabling the identification of frailty and muscle weakness for personalized interventions, such as physical training.^14^ The equation underscores the importance of condition-specific tools, enhancing understanding of HF's impact on physical condition and complementing discussions on HF management by highlighting the role of functional assessment in prognosis.^15^

## Future directions

Future studies should validate the equation in diverse populations and explore its prognostic value in longitudinal settings. Incorporating additional variables (nutritional status, ejection fraction, etc.) may improve predictive value. Integrating HGS into routine HF assessments, alongside tools like the 6-minute walk test, could enhance cardiovascular risk stratification and personalize care.^5^

## Conclusion

The proposed HGS reference equation for HF patients represents a significant step toward personalized functional assessment. Despite its limitations, the study's methodology and clinical relevance make it a simple and valuable tool for clinicians and educators. As HF management evolves, this equation can guide frailty screening and rehabilitation, ultimately improving patients’ functional outcomes.
